# 3-Bromopyruvate as an Alternative Option for the Treatment of Protothecosis

**DOI:** 10.3389/fphar.2018.00375

**Published:** 2018-04-19

**Authors:** Tomasz Jagielski, Katarzyna Niedźwiecka, Katarzyna Roeske, Mariusz Dyląg

**Affiliations:** ^1^Department of Applied Microbiology, Institute of Microbiology, University of Warsaw, Warsaw, Poland; ^2^Department of Genetics, Institute of Genetics and Microbiology, University of Wroclaw, Wroclaw, Poland

**Keywords:** *Prototheca* spp., 3-bromopyruvate, amphotericin B, synergism, cytotoxicity

## Abstract

*Protothecosis* is an unusual infection of both humans and animals caused by opportunistically pathogenic microalgae of the genus *Prototheca*. Until now, no standardized treatment protocols exist for the protothecal disease, boosted by a remarkable resistance of *Prototheca* spp. to a wide array of antimicrobial agents currently available in clinical use. Consequently, there is an urgent need for new effective drugs against *Prototheca* algae. In this study, the anti-*Prototheca* activity of 3-bromopyruvate (3BP), either alone or in combination with amphotericin B (AMB) was assessed *in vitro*, as well as the cytotoxicity of 3BP toward the bovine mammary epithelial cells and murine skin fibroblasts. The mean minimum inhibitory concentrations (MIC) and minimum algaecidal concentrations (MAC) were 0.85 ± 0.21 and 2.25 ± 0.54 mM for *Prototheca wickerhamii*, 1.25 ± 0.47 and 4.8 ± 1.03 mM for *Prototheca blaschkeae*, and 1.55 ± 0.69 and 5.6 ± 1.3 mM for *Prototheca zopfii* gen. 2, respectively. For all *Prototheca* strains tested, a synergistic interaction between 3BP and AMB was observed, resulting in about 4-fold reduction of their individual MICs, when used together. The elevated content of intracellular glutathione (GSH) was associated with a decreased susceptibility to 3BP. Both epithelial and fibroblast cells retained high viability upon treatment with 3BP at concentrations equivalent to the highest MIC recorded (3 mM) and 10-fold higher (30 mM), with the mean cell viability exceeding 80%, essentially the same as for the untreated cells. The results from these *in vitro* studies emphasize the high activity of 3BP against the *Prototheca* algae, its synergistic effect when used in combination with AMB, and the safety of the drug toward the tested mammalian cells. Along with the advantageous physico-chemical and pharmacokinetic properties, 3BP may be considered an effective and safe novel agent against the protothecal disease.

## Introduction

*Prototheca* are unicellular, yeast-like microalgae, closely related to the algal genus *Chlorella*, albeit lacking chlorophyll, and thus unable to photosynthesize (Jagielski and Lagneau, 2007). *Prototheca* are the only plant species infectious to mammals, including humans. Of the eight currently recognized species, five have been implicated in protothecal pathologies, with *P. wickerhamii* and *P. zopfii* being the most prominent causative agents of human and animal disease, respectively (Jagielski and Lagneau, 2007). Whereas, human *protothecosis* is a rare disease, with a total of 160 cases reported worldwide by 2011 (Todd et al., 2012), protothecal *mastitis*, caused mainly by *P. zopfii* and, to a lesser extent, by *P. blaschkeae* (Jagielski and Lagneau, 2007; Jagielski et al., 2012) is an escalating health and economic problem on dairy farms (Marques et al., 2006; Jagielski and Lagneau, 2007; Lassa et al., 2011; Krukowski et al., 2013). Noteworthy, *P. zopfii*, present in milk, may resist high-temperature treatments, including short-time pasteurization process (HTST) which may be hazardous to human health (Lassa et al., 2011).

Until now, no standardized treatment protocols exist for protothecal infections. Of major concern is low susceptibility and frequent resistance of *Prototheca* algae to a broad range of antimicrobials currently employed in human and veterinary medicine (Buzzini et al., 2008b; Tortorano et al., 2008; Sobukawa et al., 2011; Gao et al., 2012; Jagielski et al., 2012; Ramírez et al., 2016). Furthermore, for those drugs which are available, there is often a lack of correlation between *in vitro* susceptibility results and clinical (*in vivo*) response (Tortorano et al., 2008; Sobukawa et al., 2011). Consequently, treatment of protothecosis is largely empirical with poorly predictable and often unsuccessful outcomes. In the treatment of human infections, the drug of choice is often amphotericin B (AMB), which usually performs best, relative to other antifungals, upon drug susceptibility testing. However, an important disadvantage of AMB is its high cytotoxicity, which may produce serious kidney and liver failure (Laniado-Laborin and Cabrales-Vargas, 2009).

3-bromopyruvate (3BP), a structural analog of a key cellular metabolite (pyruvate) is one of the most widely studied compounds from the group of halogenated carboxylic acids. 3BP is characterized by a low molecular weight (166.9 g/mol), good water solubility, and alkylating properties. The number of reports on 3BP has been growing steadily since 2000, when its strong anti-tumor activity was first demonstrated with no or minimal side effects (Ko et al., 2001, 2004). 3BP acts in a complex manner, primarily through inhibition of the key glycolytic enzymes and those related with mitochondrial phosphorylation, including hexokinase II (HK2), glyceraldehyde 3-phosphate dehydrogenase GAPDH, pyruvate dehydrogenase (PDH), succinate dehydrogenase (SDH), phosphoglycerate kinase (PGK), and phosphokinase PK (Ko et al., 2001; Lis et al., 2016a,b; Yadav et al., 2017). In bacterial and fungal cells, 3BP has been shown to inhibit also isocitrate lyase and malate synthase, which play a pivotal role in the glyoxylate cycle and are associated with virulence (Dunn et al., 2009; Krátký and Vinšová, 2012).

Recent studies on the interaction of 3BP with various enzymes, including glycolytic ones, have shown that the main cellular target for this compound is HK2 followed by PDH, and SDH (Yadav et al., 2017). However, not the targets *per se*, but rather the rate at which they are achieved by 3BP is critical here. This, in turn, depends on how fast does 3BP reach the intracellular space. As shown for fungi and mammalian cells, the efficiency of 3BP transport into the cells is species-dependent. For instance, a much higher activity of this compound was observed against *C. neoformans* than against *Exophiala dermatitidis* or *Cryptococcus uniguttulatus* (Dyląg et al., 2013). The same contrasting effect was reported for multiple myeloma (MM) and peripheral blood mononuclear cells (PMBC), respectively (Majkowska-Skrobek et al., 2014). The yeast-like *Prototheca* algae are normally aerobic and possess all the enzymes, mentioned above, potentially involved in the cellular oxidation (Jagielski et al., unpublished data). These enzymes may also here act as molecular targets for 3BP.

Yet, the precise mechanism of action of 3BP has not been fully elucidated. Previous studies have reported various levels of 3BP activity against fungal species, including *Cryptococcus neoformans* (Dyląg et al., 2013, 2014). More recently, 3BP has been demonstrated as having antiprotozoal activity. Its effect against *Toxoplasma gondii* was further potentiated when used in conjunction with atovaquone (de Lima et al., 2015). Noteworthy, the wide biological activity of 3BP was coupled with a lack or negligible toxicity toward healthy mammalian cells, as shown in clinical studies and reviewed in the literature (Ko et al., 2001, 2004; Azevedo-Silva et al., 2016; Lis et al., 2016a).

The purpose of this study was to assess the *in vitro* efficacy of 3BP against *Prototheca* algae, represented by various strains of clinical and environmental origin. A combined effect of 3BP and AMB was also evaluated. In addition, the drug safety profile of 3BP was assessed by cytotoxicity assays with murine fibroblast and bovine mammary epithelial cell lines. Finally, the major route of 3BP transport into the *Prototheca* cells was investigated as well as the association between susceptibility to 3BP and intracellular glutathione (GSH) concentration. This was justified by the fact that the biological activity of 3BP had previously been linked to its uptake (accumulation) and natural GSH concentration inside the cells (Dyląg et al., 2013).

This study demonstrates a potential of 3BP as a highly effective agent against *Prototheca* spp. with minimal toxicity against host tissues.

## Materials and methods

### Chemicals

All chemicals were, if not stated otherwise, purchased from Sigma-Aldrich (USA) and were of analytical grade. The stock solutions of 3BP and AMB were prepared in water and dimethyl sulfoxide (DMSO), respectively. Working solutions of AMB were prepared in water:DMSO (80:20) immediately before use.

### Strains

Thirty strains of *Prototheca* spp. were used in this study (Table 1). Within this number were 10 strains of *Prototheca wickerhamii* (Tubaki & Soneda), 10 strains of *Prototheca blaschkeae* (Möller, Hensel, Baumann & Truyen), 9 strains of *Prototheca zopfii* (Krüger) genotype 2, and one strain of *Prototheca zopfii* var. *hydrocarbonea* (Kocková-Kratochvilová & Havelkova). All *Prototheca* strains are part of the Department of Applied Microbiology, Faculty of Biology, University of Warsaw's culture collection. The reference strains of *Candida krusei* (ATCC 6258) and *Candida albicans* (ATCC 90028) from the American Type Culture Collection (ATCC) were used as quality controls (QC) for drug susceptibility testing (CLSI, 2008). Working cultures of all the strains were maintained on Yeast extract Peptone Dextrose (YPD) agar (1% yeast extract, 2% peptone, 2% glucose, 2% agar; Difco, USA) medium.

**Table 1:** *Prototheca* spp. strains used in this study.

**Species**	**Strain^##^**	**Source**	**Reference**
*Prototheca wickerhamii*	ATCC 16529^T, E^^*^	Household plumbing, USA	–
*Prototheca wickerhamii*	IFM 52823^C**^	Human dermatitis, Japan	Hirose et al., 2013
*Prototheca wickerhamii*	CBS 344.82^C***^	Human dermatitis, South Africa	–
*Prototheca wickerhamii*	CBS 157.74^C***^	Human disseminated infection, New Zealand	Cox et al., 1974
*Prototheca wickerhamii*	SAG 263-11^E****^	Lichen *Xanthoria parietina*, Germany	–
*Prototheca wickerhamii*	A1^C^	Fish disseminated infection, Poland	Jagielski et al., 2017b
*Prototheca wickerhamii*	PWPL1^C^	Human neuroinfection, Poland	Żak et al., 2012
*Prototheca wickerhamii*	Pw-FR1^C†^	Human algaemia, France	Lanotte et al., 2009
*Prototheca wickerhamii*	P201/09^C, †^	Human algaemia, Malaysia	Mohd et al., 2012
*Prototheca wickerhamii*	SLO-1^C, †^	Human peritonitis, Slovakia	Sykora et al., 2014
*Prototheca zopfii* gen. 2	SAG 2021^T, C****^	Bovine mastitis, Germany	Roesler et al., 2006
*Prototheca zopfii* gen. 2^#^	POL-E^C^	Bovine mastitis, Poland	Jagielski et al., 2011
*Prototheca zopfii* gen. 2	POL-3^C^	Bovine mastitis, Poland	
*Prototheca zopfii* gen. 2	POL-7^C^	Bovine mastitis, Poland	
*Prototheca zopfii* gen. 2	POL-8^C^	Bovine mastitis, Poland	
*Prototheca zopfii* gen. 2	POL-9^C^	Bovine mastitis, Poland	
*Prototheca zopfii* gen. 2	POL-16^C^	Bovine mastitis, Poland	
*Prototheca zopfii* gen. 2	POL-17^C^	Bovine mastitis, Poland	
*Prototheca zopfii* gen. 2	POL-18^C^	Bovine mastitis, Poland	
*Prototheca zopfii* gen. 2	POL-19^C^	Bovine mastitis, Poland	
*Prototheca blaschkeae*	SAG 2064^T, C****^	Human onychomycosis, Germany	Roesler et al., 2006
*Prototheca blaschkeae*	POL-20^C^	Bovine mastitis, Poland	Jagielski et al., 2011
*Prototheca blaschkeae*	49/IV^C^	Bovine mastitis, Poland	–
*Prototheca blaschkeae*	8^C^	Bovine mastitis, Poland	
*Prototheca blaschkeae*	560^C^	Bovine mastitis, Poland	
*Prototheca blaschkeae*	BŚ^E^	Cow bedding, Poland	
*Prototheca blaschkeae*	AK1^E^	Bovine feces, Poland	
*Prototheca blaschkeae*	36	Bovine mastitis, Poland	
*Prototheca blaschkeae*	BŁ3^E^	Mud from a cowshed, Poland	
*Prototheca blaschkeae*	204090^†*E*^	Cow milk, Italy	

The strains were purchased from the following culture collections: American Type Culture Collection (ATCC), Manassas, VA, USA (*); Research Center for Pathogenic Fungi and Microbial Toxicoses, Chiba University, Chiba, Japan (**), Centraalbureau voor Schimmelcultures (CBS), Baarn, The Netherlands (***), Culture Collection of Algae at the University of Göttingen (SAG), Göttingen, Germany (****) or kindly provided by collaborating investigators, named in the Acknowledgments section of the article (^†^);

# P. zopfii gen. 2 (var. hydrocarbonea);

## *Type strains, disease-related (clinical) and –unrelated (environmental) strains were designated with the superscript letters “T”, “C”, and “E”, respectively*.

### Cell lines and culture conditions

Bovine mammary epithelial cell line (BME-UV1) was kindly provided by the Istituto Zooprofilattico Sperimentale della Lombardia e dell'Emilia Romagna, Brescia, Italy. The clonal cell line BME-UV1 was established from primary bovine epithelial cells in culture by stable transfection with a plasmid carrying the sequence of the simian virus 40 large T-antigen. It represents a valid model system to examine bovine mammary epithelial proliferation and differentiation and cell-to-cell communication (Zavizion et al., 1996). The murine fibroblast cell line L-929 was obtained from the Hirszfeld Institute of Immunology and Experimental Therapy, Polish Academy of Sciences, Wroclaw, Poland. The murine fibroblast cell line L-929 (connective tissue, mouse) clone of the parent L strain derived from normal subcutaneous areolar and adipose tissue of a 100-day-old male C3H/An mouse. Clone 929 was established (by the capillary technique for single cell isolation) from the 95th subculture generation of the parent strain (Earle et al., 1943).

Both cell lines were maintained in T-flasks incubated at 37°C in a 5% CO_2_ and 100% humidity incubator (IG150 Jouan, Spain). For BME-UV, the culture medium was a mixture of DMEM-F12 (Sigma-Aldrich, USA), RPMI-1640 (Life Technologies, USA), and NCTC-135 (Sigma-Aldrich, USA) at a ratio of 4:3:2, supplemented with 10% fetal bovine serum, lactose monohydrate (0,1%), lactalbumin enzymatic-hydrolysate (0.1%), ascorbic acid (10 mg/L), hydrocortisone (1 mg/L), L-glutathione (1.2 mM), insulin (1 mg/L), penicillin (50 IU/mL), and streptomycin (50 mg/L). For L-929 cells, the medium was RPMI-1640 with 2 mM L-glutamine (Gibco, USA) supplemented with 10% fetal bovine serum. The cells were grown until 80% confluence. For subculturing, the cells were detached from the surface of the flask with a 0.1% trypsin/EDTA solution (Gibco, USA), transferred to 50-mL Falcon tubes, centrifuged (8 min, 1,600 rpm, 4°C), and resuspended in a fresh culture medium. The number of viable cells was determined using trypan blue staining and cell counting in a hemocytometer chamber under light microscope (IX70, Olympus, Japan).

### Minimal inhibitory concentration (MIC) and minimal algaecidal concentration (MAC) determination

**Figure 1:**
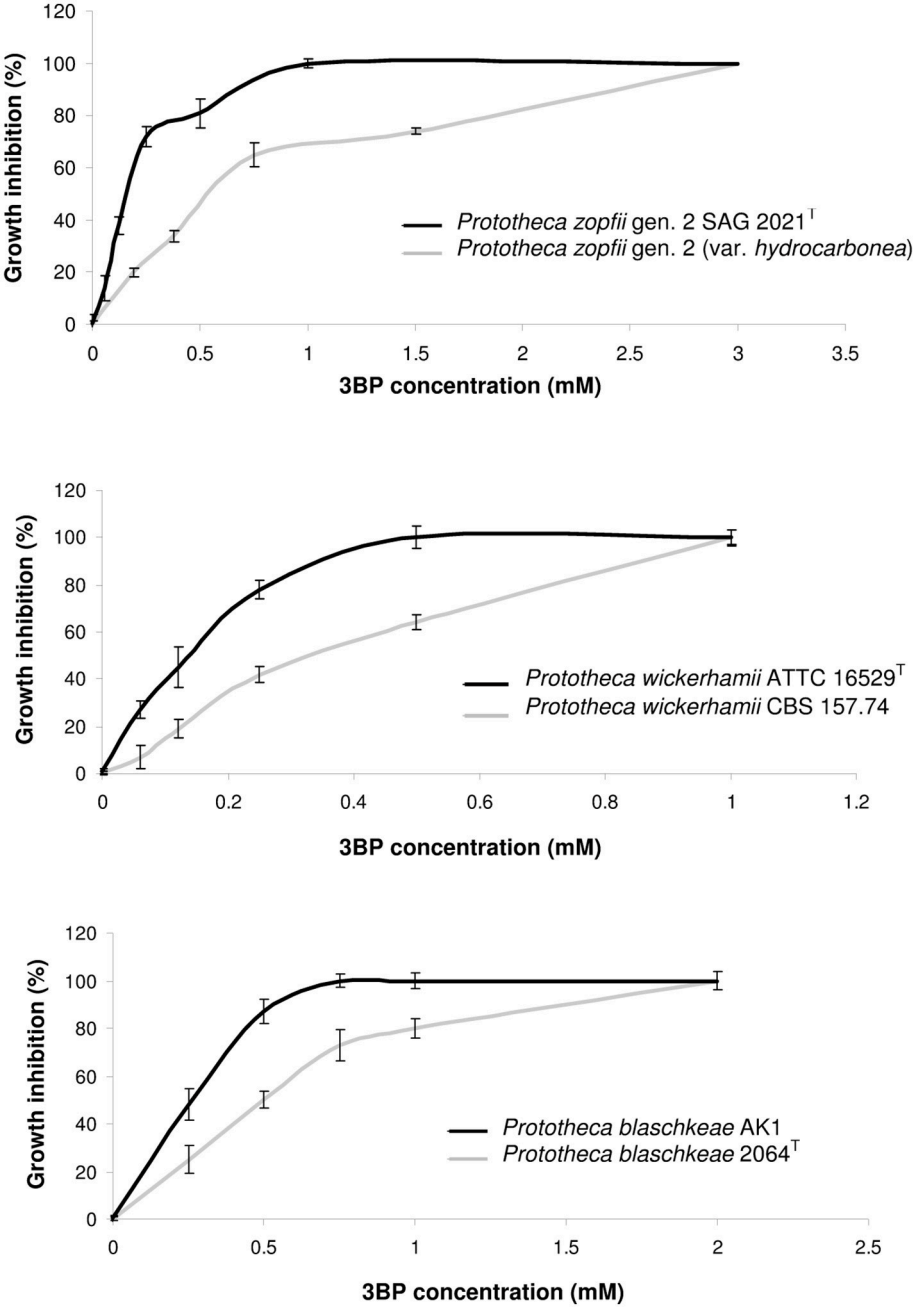
Dose-dependent inhibition of growth and proliferation of cells in case of different *Prototheca* spp. strains.

In the absence of universally accepted procedures or interpretative criteria specific for *Prototheca* spp., determination of MICs of 3BP and AMB was performed according to the broth microdilution method, in 96-well microtiter plates (NuncTM, Denmark), strictly following the Clinical Laboratory Standards Institute (CLSI) protocol (M27-A3) for drug susceptibility testing of yeast-like fungi (CLSI, 2008). The only modification was that the pH of the RPMI-1640 (Sigma-Aldrich, USA) medium was adjusted to 5.5 instead of 7.0 ± 0.1. This modification was due to the best penetration of 3BP, at that pH, as evidenced and explained elsewhere (Azevedo-Silva et al., 2015; Casal et al., 2016). The MIC was defined as the lowest drug concentration that completely inhibited algal growth upon OD measurement at 600 nm in the Asys UVM 340 (Biochrom Ltd., UK) microplate reader. The MAC values were determined as described earlier (Espinel-Ingroff et al., 2002; Pillai et al., 2005). Briefly, after MIC determination, 100-μL samples taken from appropriate microwells were spread across the surface of the YPD medium. The MAC was defined as the lowest drug concentration at which ~99.5–99.9% of cells of algal population were killed, when compared to growth control (drug-free medium; Pillai et al., 2005). For the MIC/MAC determination, the following drug concentrations were used: 0.06, 0.09, 0.12, 0.19, 0.25, 0.38, 0.5, 0.75, 1.0, 1.5, 2.0, 3.0, 4.0, 6.0, 8.0, 12, and 16 mM—for 3BP, and 0.06, 0.09, 0.12, 0.19, 0.25, 0.38, 0.5, 0.75, 1.0, 1.5, 2.0, 3.0, and 4.0 mg/L—for AMB). Both the MIC/MAC values as well as the percentage of growth inhibition (%) were read after 48-h incubation at 35°C. The *in vitro* dose-dependent 3BP toxicity against different strains of *Prototheca* spp. was assessed based on the spectrophotometric measurements of optical density (OD) values, upon microdilution assay, and was expressed as the percentage of growth inhibition at different 3BP concentrations tested, with reference to control (3BP-free sample; Figure 1). All tests were performed in triplicate. Only if two replications showed identical results was the strain given the final MIC/MAC value of the drug tested.

### Synergy assessment

**Figure 2:**
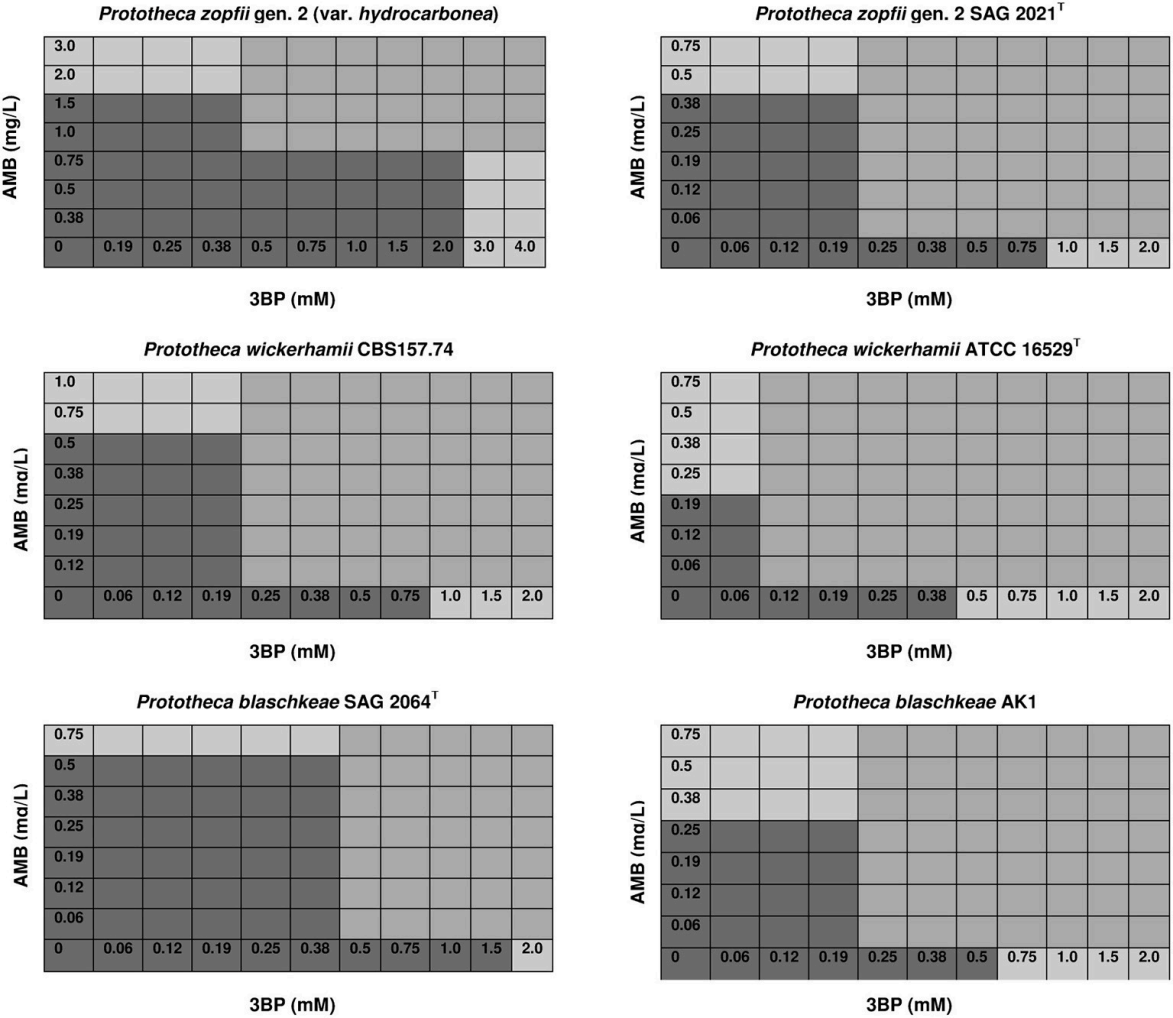
Effective concentrations of 3BP and AMB used in combination that inhibit growth and proliferation of cells of different *Prototheca* spp. (mod-gray color) and not effective when used solely (dark gray color)—selected data.

To assess the interactions between 3BP and AMB, 2D checkerboard assays were performed in RPMI-1640 medium, in 96-well microtiter plates (Nunc^TM^, Denmark), following the standard protocol (Hindler and Munro, 2010). Briefly, a total of 100 μL containing 50 μL of a 2-fold concentrated drug (3BP or AMB) solution, diluted in RPMI-1640, was dispersed into each well of the microtiter plate. Each well was then inoculated with 100 μL of the algal suspension, in RPMI-1640, equivalent to a 0.5 McFarland turbidity standard (*ca*. 1.0 × 10^6^ CFU/ml). Thus, a concentration gradient of 3BP 0.06–4 mM (10.44–667.84 mg/L) was generated along the abscissas, and that of AMB 0.06–4.0 mg/L (0.07–4.32 × 10^−3^ mM) along the ordinates (Figure 2). Plates were incubated at 35°C for 48 h under aerobic conditions. The concentrations of compounds were chosen according to the preliminary results for *Prototheca* spp. (Buzzini et al., 2008b; Jagielski et al., 2012; Niedźwiecka et al., 2016). Each time the type of interaction between 3BP and AMB was defined based on the Fractional Inhibitory Concentration Index (FICI). FICI values were calculated as follows: FICI = (MIC of 3BP in combination with AMB/MIC of 3BP alone) + (MIC of AMB in combination with 3BP/MIC of AMB alone). Interactions between drugs were defined as [S] synergistic (FICI ≤ 0.5), [A] additive (0.5 < FICI ≤ 1), [N] neutral (1 < FICI ≤ 2), or [An] antagonistic (2 < FICI) (Hindler and Munro, 2010). In order to better illustrate the type of interaction between the two compounds tested, isobologram analysis (Barrera et al., 2005; Chou, 2006) was performed for the most and least susceptible to 3BP strains of each *Prototheca* species.

### Transport of 3BP into the *Prototheca* cells

The uptake of the [^14^C]-labeled 3BP was carried out by the modified method reported for L-lactate transport (Casal et al., 1999). For each *Prototheca* spp. strain, a culture (10 mL) was grown in liquid synthetic dextrose (SD) medium: 0.67% yeast nitrogen base w/o amino acids (Difco, USA), 2% glucose (POCH, Poland) and incubated at 28°C until the optical density at 600 nm reached *ca*. 0.5. Cultures in exponential phase of growth were centrifuged (5 min, 5,000 rpm, 4°C) and washed two times with sterile ice-cold deionized water. The algal cells were resuspended in 20 mL of fresh SD medium with glucose replaced with lactic acid as a sole carbon source, and further incubated for 4 h. After incubation cultures were centrifuged (5 min, 5,000 rpm, 4°C) and the pellets were washed twice and resuspended in 0.1 M phosphate buffer (pH 5.0) to a final concentration of 200 mg/mL (wet weight). Subsequently, 90 μL of samples were mixed with 10 μL of cold [^14^C]-labeled 3BP of different concentrations, so that the following final concentrations were: 0.1, 0.2, 0.5, 1.0, 2.0, and 3.0 mM. After 1 min incubation at 30°C the reaction was stopped by the addition of 100 μL of ice-cold deionized water. The samples were mixed and centrifuged (5 min, 14,000 rpm, 4°C). To each pellet, 1 mL of scintillation fluid was added and the cell suspension was filtrated on nitrocellulose Whatman filters using a vacuum filtration box (Hoefer, USA). The radioactivity of the protothecal cells, harvested in the scintillation fluid (Perkin-Elmer, USA), was measured in a liquid scintillation counter (Beckman LS100, USA). The results were normalized to the dry mass of cells. All charts and calculations were made using the GraphPad Prism 5.01 program (GraphPad Software, USA).

### Determination of glutathione concentration

The concentration of intracellular glutathione was determined for selected *Prototheca* spp., whose cultures were maintained in RPMI-1640 medium at 35°C with shaking, until the optical density at 600 nm reached 1.5–1.8. The GSH level was assayed using the boiling buffered ethanol procedure for quantitative metabolite extraction and Ellman's test, as described elsewhere (Ellman, 1959; Gonzalez et al., 1997; Lis et al., 2012; Dyląg et al., 2013). GSH concentration was given in μM per a million (10^6^) of cells. All tests were done in triplicate at least.

### Cytotoxicity assay

For the 3BP cytotoxicity study, cells of both lines were seeded in 96-well microtiter plates and cultured until 100% confluent (*ca*. 1.0 × 10^5^ cells/well). The medium was refreshed and 3BP, dissolved in 0.1 M phosphate buffer pH 6.5 without sodium ions (or the buffer alone in a control well), was added to the final concentrations, incrementally increasing by 10-fold from 0.03 to 30 mM.

The cytotoxicity of 3BP was evaluated with the LIVE/DEAD Viability/Cytotoxicity Kit for mammalian cells (Invitrogen, USA). The kit discriminates live from dead cells by staining live cells with green-fluorescent calcein-AM to indicate intracellular esterase activity and dead cells with red-fluorescent ethidium homodimer-1 (EthD-1) to indicate loss of plasma membrane integrity. Assessment of cell viability was performed according to the manufacturer's instructions. Briefly, the monolayers were exposed, post 24-h incubation, to 100 μL of 8 μM calcein-AM and 4 μM EthD-1 solution in RPMI-1640 medium per well and incubated in 5% CO_2_ at 37°C for 30 min. Wells containing all the cells dead (treated with 70% ethanol for 30 min) and all the cells alive (non-treated), stained with single-dye solutions, served as negative and positive controls, respectively. Fluorescence of the calcein-AM and Eth-D1 was measured using an Infinite M200 Pro microplate reader equipped with a fluorometer (Tecan, Austria) with the excitation/emission filter pairs 485/530 and 530/645, respectively. Background fluorescence, measured in wells containing both dyes, but not cells, was subtracted from all values before calculating mean fluorescence for the groups.

Percentage cell viability was calculated using the following formula: cell viability [%] = (fluorescence of drug-treated (non-treated) sample/fluorescence of positive-control sample) × 100. Three independent experiments were carried out, for a total of three repeats for each 3BP concentration and for the controls. The results were expressed as means ± standard deviation (SD).

### Statistical analysis

The results of susceptibility tests and determination of intracellular glutathione concentration were presented as the mean ± SD from of at least three independent experiments. In the case of transport assay statistical significance was assessed by one-way analysis of variance (ANOVA) using GraphPad Prism 5.01 program (GraphPad Software, USA).

Statistical differences between cell viability levels corresponding to different 3BP concentrations were analyzed by the repeated measures ANOVA with the sphericity assumption upon the Mauchly's test. *Post-hoc* pairwise comparisons were made using the Šidák correction. Differences in cell viability between the two cell lines used, at successive concentrations of 3BP, were assessed by the Wilcoxon signed rank test. All analyses were performed using IBM SPSS Statistics ver. 23 (IBM Corp., Armonk, NY, USA). Statistical significance was set at *P* ≤ 0.05.

## Results

The MIC and MAC values of 3BP determined *in vitro* for 30 *Prototheca* strains tested are presented in Table 2. The most susceptible toward 3BP were strains of *P. wickerhamii*. For these strains, the MICs were either 0.5, 0.75, or 1.0 mM. In contrast, *P. zopfii* gen. 2 and *P. blaschkeae* strains were significantly less sensitive, with their MICs ranging from 1.0 to 3.0 and 0.75–2.0 mM, respectively. The inter- and intra-species differences in the susceptibility to 3BP were apparent upon inspection of dose-dependent curves (Figure 1). The algaecidal capacity of 3PB was ranked in the same order as its algaestatic potential, with *P. wickerhamii* strains and *P. zopfii* gen. 2, showing the lowest and highest MAC values, accordingly (1.5–3 vs. 4–8 mM). For all *Prototheca* strains, the MAC values were ~3–4 times higher than their MIC values (Table 2). In general, AMB exhibited a higher activity against *P. wickerhamii* and *P. blaschkeae* than against *P. zopfii*. The MICs of the former two species were in the range of 0.25–0.75 and 0.38–0.75 mg/L, respectively. In contrast, *P. zopfii* had their AMB MICs between 0.5 and 2.0 mg/L. The mean MIC value of AMB for all *Prototheca* strains equaled 0.67 × 10^−3^ mM (0.622 mg/L) and was *ca*. 1821-(327)-fold lower than the mean MIC value of 3BP (1.22 mM or 203.7 mg/L; Tables 2, 3).

**Table 2:** Biological activity of 3BP against *Prototheca* spp.

**Tested strains^#^**	MIC^*^ range	**Mean**	MAC^*^ range	**Mean**
*Prototheca blaschkeae* (6C+4E)	0.75–2.0 (125.2–333.9)	1.25 ± 0.47 (208.7 ± 78.5)	4.0–6.0 (667.8–1001.8)	4.8 ± 1.03 (801.4 ± 171.9)
*Prototheca wickerhamii* (8C+2E)	0.5–1.0 (83.5–166.9)	0.85 ± 0.21 (141.9 ± 35.1)	1.5–3.0 (250.4–500.9)	2.25 ± 0.54 (375.7 ± 90.2)
*Prototheca zopfii* gen. 2 (10C)	1.0–3.0 (166.9–500.9)	1.55 ± 0.69 (258.8 ± 115.2)	4.0–8.0 (667.8–1335.7)	5.6 ± 1.3 (934.9 ± 217.1)

# No. of isolates are given in brackets; C, clinical origin; E, environmental origin;

* *MIC, Minimal Inhibitory Concentration; MAC, Minimal Algaecidal Concentration; all values given in mM and mg/L (in brackets)*.

**Table 3:** Determination of the type of interaction between 3BP and AMB.

**No. of isolates^#^**			**MIC^##^ in mg/L (mM)**				**FICI^###^**
***P.z*. (10)**	***P.b*. (10)**	***P.w*. (10)**	**3BP alone**	**AMB alone**	**3BP in assoc. with AMB**	**AMB in assoc. with 3BP**	
3	1	0	333.92 (2.0)	0.75 (0.81 × 10^−3^)	83.48 (0.5)	0.063 (0.068 × 10^−3^)	0.21 [S]
0	1	0	250.44 (1.5)	0.5 (0.54 × 10^−3^)	20.87 (0.125)	0.09 (0.097 × 10^−3^)	0.26 [S]
2	1	2	166.96 (1.0)	0.5 (0.54 × 10^−3^)	41.74 (0.25)	0.063 (0.068 × 10^−3^)	0.38 [S]
0	1	0	333.92 (2.0)	0.38 (0.41 × 10^−3^)	83.48 (0.5)	0.063 (0.068 × 10^−3^)	0.42 [S]
2	1	2	166.96 (1.0)	0.75 (0.81 × 10^−3^)	41.74 (0.25)	0.125 (0.135 × 10^−3^)	
0	0	1	83.48 (0.5)	0.25 (0.27 × 10^−3^)	20.87 (0.125)	0.063 (0.068 × 10^−3^)	0.50 [S]
0	1	2	125.22 (0.75)	0.38 (0.41 × 10^−3^)	41.74 (0.25)	0.063 (0.068 × 10^−3^)	
0	1	2	166.96 (1.0)	0.38 (0.41 × 10^−3^)	41.74 (0.25)	0.125 (0.135 × 10^−3^)	0.58 [A]
0	1	0	125.22 (0.75)	0.38 (0.41 × 10^−3^)	41.74 (0.25)	0.125 (0.135 × 10^−3^)	0.66 [A]
1	0	0	500.88 (3.0)	2.0 (2.16 × 10^−3^)	83.48 (0.5)	1.0 (1.08 × 10^−3^)	0.67 [A]
1	0	0	250.44 (1.5)	0.75 (0.81 × 10^−3^)	125.22 (0.75)	0.125 (0.135 × 10^−3^)	
1	0	0	166.96 (1.0)	1.5 (1.62 × 10^−3^)	63.44 (0.38)	0.5 (0.54 × 10^−3^)	0.71 [A]
0	1	0	166.96 (1.0)	0.5 (0.54 × 10^−3^)	83.48 (0.5)	0.125 (0.135 × 10^−3^)	0.75 [A]
0	0	1	83.48 (0.5)	0.38 (0.41 × 10^−3^)	41.74 (0.25)	0.125 (0.135 × 10^−3^)	0.83 [A]
0	1	0	250.44 (1.5)	0.5 (0.54 × 10^−3^)	125.22 (0.75)	0.25 (0.27 × 10^−3^)	1.00 [A]

# No. of isolates are given in brackets; P.b., Prototheca blaschkeae; P.w., Prototheca wickerhamii; P.z., Prototheca zopfii gen. 2;

## MIC, Minimal Inhibitory Concentration values determined according to microdilution assay;

### *FICI, Fractional Inhibitory Concentration Index; [S], hipersynergism; [A], additive synergism*.

**Figure 3:**
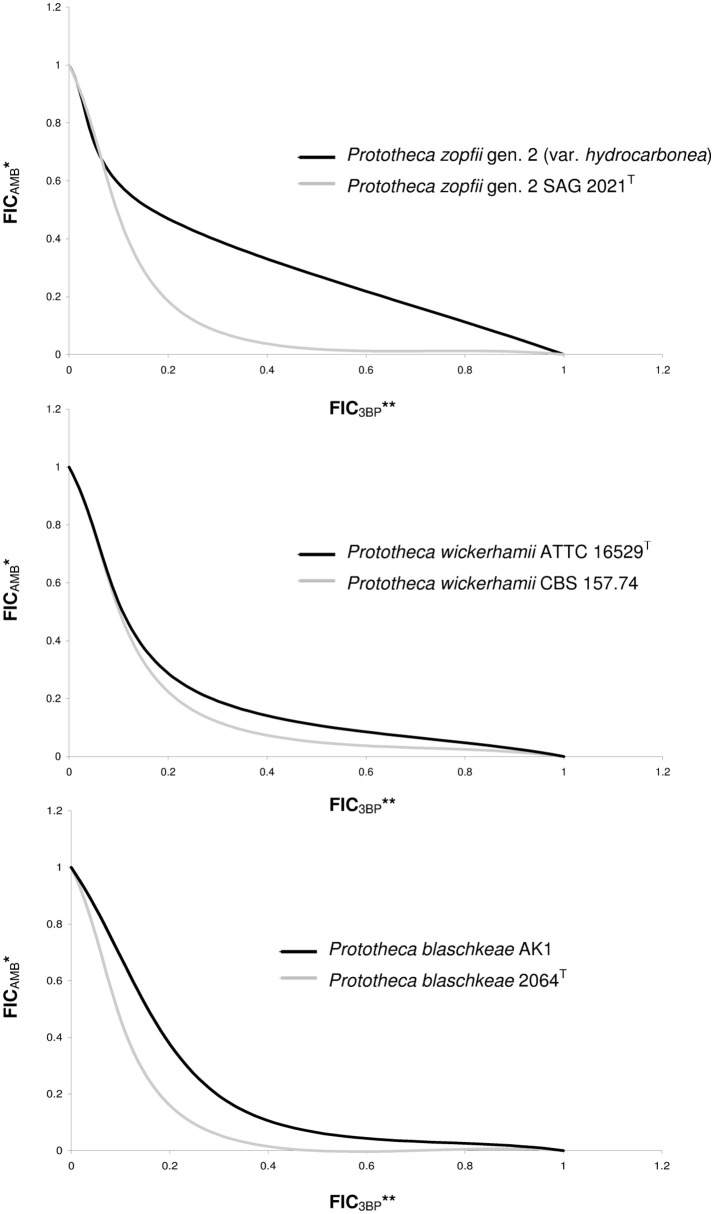
Isobolograms showing synergistic or additive types of interactions between AMB and 3BP. *FIC_AMB_, Fractional Inhibitory Concentration of AMB, defined as the ratio of the MIC of AMB in association with 3BP to the MIC of AMB tested alone; **FIC_3BP_, Fractional Inhibitory Concentration of 3BP, defined as the ratio of the MIC of 3BP in association with AMB to the MIC of 3BP tested alone; FIC values determined based on 2D checkerboard assay.

The MIC values of 3BP and AMB, when used in combination, were considerably lower than the MIC values of each drug used separately (Figure 2). The mean MIC values of 3BP and AMB used in combination with each other were ~3.6- and 4.4-fold lower, respectively than the mean MICs of these corresponding drugs used alone (given above). The combined effect of the two compounds was hipersynergistic for 6 *P. blaschkeae* strains, 7 *P. wickerhamii* strains, and 7 *P. zopfii* strains, with FICIs calculated at 0.21–0.50, 0.38–0.5, and 0.21–0.42, accordingly. For the remaining 10 *Prototheca* strains, additive synergism was observed, with FICIs ranging from 0.58–1.0 (*P. blaschkeae*), 0.58–0.83 (*P. wickerhamii*), and 0.67–0.71 (*P. zopfii*) (Table 3). Hipersynergism or additive synergism between 3BP and AMB was also evident upon isobologram analysis. For *P. blaschkeae* and *P. wickerhamii* strains, the isobole invariably showed synergism, while for one *P. zopfii* gen. 2 strain (least 3BP-sensitive), an additive effect was observed (Figure 3).

**Figure 4:**
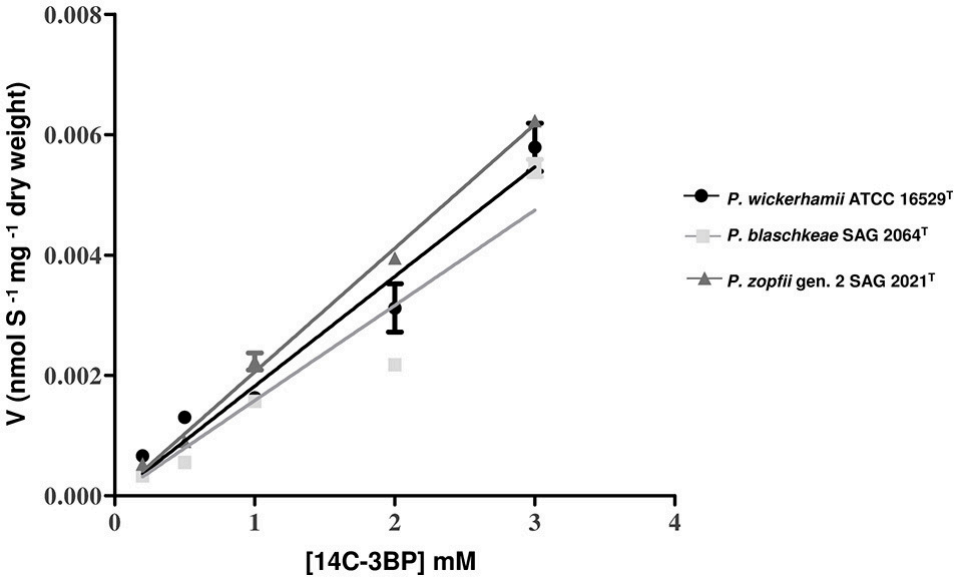
The uptake kinetics of [^14^C]-labeled 3BP (substrate concentration range: 0–3 mM) in different *Prototheca* spp. strains.

To investigate whether the observed differences in the susceptibilities to 3BP between the *Prototheca* strains are related to different cell membrane permeabilities, the [^14^C]-labeled 3BP uptake assays were performed on three *Prototheca* species under the study (i.e., *P. wickerhamii* ATCC 16529^T^, *P. blaschkeae* SAG 2064^T^, *P. zopfii* gen. 2 SAG 2021^T^). As shown in Figure 4, the uptake velocity of the [^14^C]-labeled 3BP was very similar for all three *Prototheca* species. Generally the velocity of 3BP uptake into the *Prototheca* cells was directly proportional to the drug concentration in the cell environment. At the highest concentration employed (3 mM), the uptake velocity was equal to 0.005444, 0.00579, and 0.00623 nmoles × mg^−1^ × dry weight for *P. blaschkeae, P. wickerhamii*, and *P. zopfii*, respectively (Figure 4).

**Figure 5:**
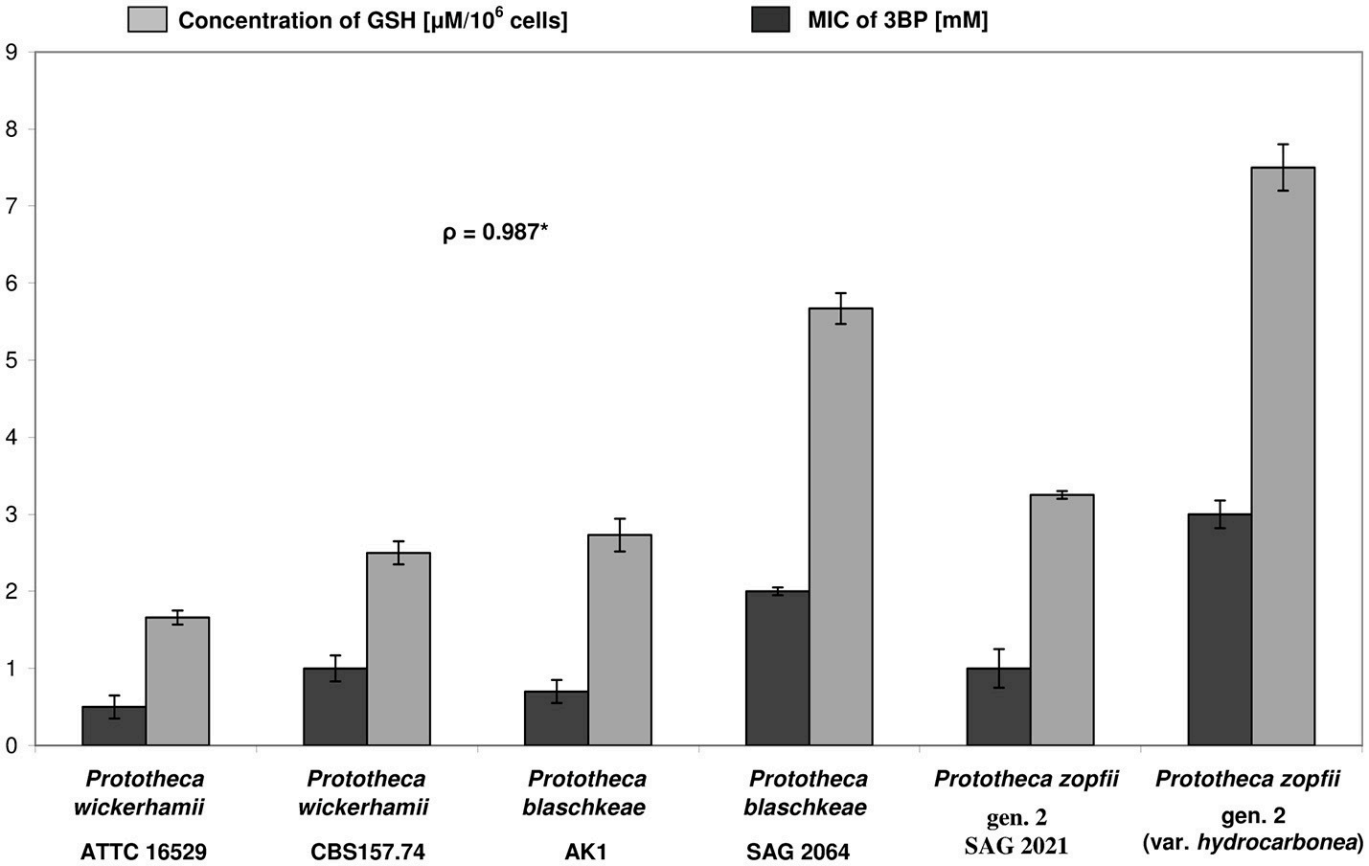
Differences in susceptibility (MIC values) toward 3BP (evaluated by the microdilution assay) and in concentrations of intracellular glutathione (GSH) between different strains of *Prototheca* microalgae. ^*^Pearson correlation coefficient; All tests were repeated at least three times.

The basis for the inter-species differences in the 3BP susceptibility was also investigated by comparing the intracellular GSH concentration. Two strains of each species, with extreme MIC and MAC values of 3BP were tested. The highest GSH level was observed for *P. zopfii* gen. 2 (var. *hydrocarbonea*) and equaled 7.5 μM per 10^6^ cells. Interestingly, this strain exhibited also the highest MIC (3.0 mM) and MAC (8.0 mM) values of 3BP among all *Prototheca* strains. For *P. zopfii* gen. 2 SAG 2021^T^ (type strain, MIC, 1.0 mM; MAC, 4.0 mM), the intracellular GSH concentration was clearly lower (3.25 μM per 10^6^ cells). Among *P. wickerhamii* strains, those most and least susceptible to 3BP (MICs, 0.5 vs. 1.0 mM; MACs, 1.5 vs. 3.0 mM) had their GSH content of 1.66 and 2.5 μM per 10^6^ cells, respectively. Of the *P. blaschkeae* strains, those with the highest and lowest MIC and MAC values of 3BP (MICs, 0.75 vs. 2.0 mM; MACs, 4.0 vs. 6.0 mM) exhibited GSH concentrations at 2.73 and 5.67 μM per 10^6^ cells, respectively (Figure 5). Overall, the mean intracellular GSH content in *P. zopfii* was 1.3-fold higher than that in *P. blaschkeae*, while the mean intracellular GSH content in *P. blaschkeae* was twice that in *P. wickerhamii*. All these results were obtained from the three independent replicates and included SD values (Figure 5, data not shown in the text).

**Figure 6:**
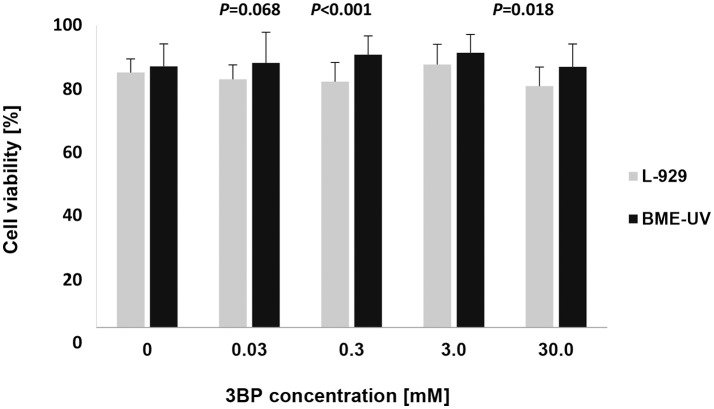
Viability of cultured murine fibroblasts (L-929) and bovine mammary epithelial cells (BME-UV1) determined using LIVE/DEAD Viability/Cytotoxicity assay. Gray and black bars represent viability of L-929 and BME-UV cells, respectively. Data are expressed as mean percentage ± SD of six experiments, each performed in triplicate.

The cytotoxicity assay demonstrated that 3BP, at a concentration equivalent to the highest 3BP MIC value (3.0 mM) and a concentration 10-fold higher (30 mM), did not affect the viability of both murine fibroblast and bovine epithelial cells, compared to the untreated cells (Figure 6). For both cell lines, the overall cytotoxicity results were statistically significant (*P* < 0.05), yet the pairwise comparisons failed to reveal any significant differences between the concentrations used. Murine fibroblasts were slightly more susceptible to 3BP than the bovine mammary epithelial cells (mean cell viability, 80 ± 0.06–87 ± 0.07 vs. 86 ± 0.07–91 ± 0.06). The differences in cell viability between the two cell lines were statistically significant at concentrations of 0.03, 0.3, and 30 mM (Figure 6).

## Discussion

Treatment of protothecosis remains a serious challenge in both human and animal medicine. An important reason for this is a remarkable resistance of *Prototheca* spp. to a wide array of antimicrobial agents currently available in clinical use (Buzzini et al., 2008b; Tortorano et al., 2008; Sobukawa et al., 2011; Jagielski et al., 2012). Therefore, there is a constant need for new effective drugs against *Prototheca* algae. In a quest for such agents, various chemical compounds have been investigated including plant-derived flavonoids, polyphenols, essential oils, hormones (Turchetti et al., 2005; Buzzini et al., 2008a; Tortorano et al., 2008; Wawron et al., 2013; Bouari et al., 2014; Grzesiak et al., 2016), animal-derived lactoferrin (Kawai et al., 2007), iodine-based products (Cunha et al., 2010; Salerno et al., 2010; Lassa et al., 2011; Răpuntean et al., 2015; Jagielski et al., 2017a), guanidine (Alves et al., 2017) or silver nanoparticles (Jagielski et al., unpublished data). Although the results for some of these compounds are promising, their use seems to be largely limited to external and disinfectant (antiseptic) rather than therapeutic applications.

In this study, similarly to what was observed in different fungal species (Dyląg et al., 2013), a high, yet variable, activity of 3BP toward *Prototheca* spp. was demonstrated. The MIC and MAC values for the *Prototheca* strains tested ranged from 0.5 to 3.0 mM and from 1.5 to 8.0 mM, respectively. The most susceptible species was *P. wickerhamii*, with the mean MICs and MACs of 0.85 ± 0.21 and 2.25 ± 0.54 mM, respectively, followed by *P. blaschkeae* (1.25 ± 0.47; 4.8 ± 1.03 mM) and *P. zopfii* gen. 2 (1.55 ± 0.69; 5.6 ± 1.3 mM). The same susceptibility order was also established for AMB, with the mean MIC values of 0.46 ± 0.16, 0.5 ± 0.14, and 0.9 ± 0.47 mM, accordingly. These results are consistent with previously published findings. Firstly, the inter-species differences in the 3BP activity has been observed for yeast of the *Cryptococcus* genus (Dyląg et al., 2013, 2014). Secondly, susceptibility results for AMB were in close agreement with those previously described (Marques et al., 2006; Tortorano et al., 2008; Sobukawa et al., 2011; Gao et al., 2012; Jagielski et al., 2012, 2017a; Wawron et al., 2013). Thirdly, similar to earlier studies, *P. zopfii* gen. 2 displayed the most drug-resistant phenotype among the *Prototheca* spp. (Jagielski et al., 2012, 2017a).

Differences in the susceptibility to 3BP have been linked to a differentiated transport of 3BP into the cells and the intracellular content of glutathione (Dyląg et al., 2013, 2014).

To test if similar relationships exist with regard to the *Prototheca* spp. [^14^C]-labeled 3BP and GSH concentration assays were performed, as it was accomplished for *C. neoformans* and other fungi (Dyląg et al., 2013).

In contrast to findings established for fungi (Lis et al., 2012; Dyląg et al., 2013), the uptake of radiolabeled 3BP by protothecal cells was shown to be species non-specific. Moreover, it was linearly dependent on concentration, suggesting to be non-saturable (not resembling the typical Michaelis-Menten curve) and not influenced by the membrane permeability properties (Figure 4). This speaks in favor of the absence of any membrane transporters specifically involved in the uptake of 3BP into the protothecal cells. This is in contrast to findings in fungi and mammalian cells, for which such membrane proteins have been described (Lis et al., 2012; Dyląg et al., 2013; Majkowska-Skrobek et al., 2014). This may be relevant with respect to the mode of action of 3BP in *Prototheca* cells. Due to the lack of transporters specifically convoying 3BP, the compound penetrates into the cells through simple diffusion and quickly achieves lethal concentrations. Also, as demonstrated earlier, 3BP cannot be actively removed from the cell, as it is not a substrate for ABC transporter efflux pumps (Lis et al., 2012).

The lack of differences in the 3BP transport between different *Prototheca* spp. prompted to examine the concentration of GSH inside the algal cells. As it was shown previously for different fungi, the natural level of GSH has been associated with the differences in the sensitivity toward 3BP (Dyląg et al., 2013). Moreover, the stimulating effect of 3BP, used in sub-MIC concentrations, on the expression of genes involved in GSH metabolism has been shown (Niedźwiecka et al., 2016). Finally, the decrease of intracellular GSH levels over the time in the presence of 3BP was demonstrated (Niedźwiecka et al., 2016).

Suspectedly, an interspecies variation (or between different strains) was observed in the intracellular GSH concentration. The natural (not induced by drug) different concentrations of intracellular GSH was associated with susceptibility to 3BP in all three *Prototheca* species assayed. In general, the higher the GSH content was, the less susceptible was the algal strain. This finding is consistent with what had previously been observed for fungi. GSH is a well-known natural antioxidant whose functions include countering oxidative stress and heavy metal, herbicide, and xenobiotic detoxification (Pócsi et al., 2004).

Apart from the evaluation of the 3BP efficacy toward *Prototheca* algae, we were eager to see how would it perform in conjunction with AMB, a compound of known anti-protothecal activity. AMB is one of the most powerful antifungals, with activity against a wide range of yeast and filamentous pathogenic fungi. This broad spectrum of activity, along with a low incidence of acquired resistance has earned AMB a place as a mainstay of antifungal therapy, including life-threatening systemic mycoses (Nett and Andes, 2016). An important disadvantage of the drug is its high potential toxicity, limiting dose intensity and clear-cut improvement. A possible remedy to this is to use AMB in combination with another drug, so that each drug could achieve its therapeutic effectiveness, yet having been administered at a lower dose.

Following this logic, AMB has been tested with many other drugs, and the synergistic antifungal effect has been noted when the polyene was combined with anidulafungin (Drogari-Apiranthitou et al., 2012), tetracycline (Oliver et al., 2008), rifampicin (He et al., 2017), terbinafine (Ryder and Leitner, 2001), 5-fluorocytosine, and the azoles (Odds, 1982). Although studies on drug interactions in the context of anti-*Prototheca* therapy are very few, a synergy between AMB and tetracycline has repeatedly been demonstrated since the mid-1970s (Todd et al., 2012). The reduction of the MIC of AMB against *Prototheca* spp. was also evident in the presence of rifampicin (Srimuang et al., 2000). Our results clearly demonstrated that 3BP and AMB act synergistically, allowing for about 4-fold decrease in their MICs when used together. Based on the FICI values, the combined effect of 3BP and AMB was hipersynergistic for two-thirds of the *Prototheca* strains, whereas for the remaining strains an additive synergism was noted. The synergistic interaction between the two drugs may be explained in the following way: AMB, which disrupts membrane structure through binding to the cell membrane constituent, ergosterol, accelerates the cellular uptake of 3BP, and facilitates reaching of its target molecules in the cellular milieu. However, to propose any model for AMB and 3BP interactions, precise molecular targets of the latter agent need to be disclosed.

To better assess the clinical application of 3BP, its cytotoxic capacity was evaluated on bovine mammary gland epithelium cells and murine skin fibroblasts, as the most frequently affected in protothecal infections of animals and humans, respectively. Both of the cell lines retained high viability upon treatment with 3BP at concentrations equivalent to the highest MIC recorded (3 mM) and even 10-fold higher (30 mM), with the mean cell viability exceeding 80%, almost matching the results obtained for the untreated control cells. These findings are consistent with previous studies, which show a potent anticancer (Ko et al., 2001, 2004; Azevedo-Silva et al., 2016) as well antifungal, especially anticryptococcal (Dyląg et al., 2013, 2014) activity of 3BP. This compound elicited fast toxicity toward tumor cells, while exerting no damage to healthy tissues (Ganapathy-Kanniappan et al., 2012; Kunjithapatham et al., 2013). The mechanism behind this selective effect of 3BP was explained elsewhere (Azevedo-Silva et al., 2016; Lis et al., 2016a,b). The side effects after 3BP administration were consistently absent with low-doses of 3BP such as 1.75 mM, even with systemic drug delivery strategies (Ganapathy-Kanniappan et al., 2012; Kunjithapatham et al., 2013). The minimal toxicity of 3BP noted in the present study, despite much higher concentration (30 mM) may stem from the fact that here the toxicity was tested on cell lines, not tissue sections, in contrast to the works cited above.

Considering the therapeutic potential of 3BP, it is noteworthy that this pyruvate analog has a short half-life of <80 min at physiological temperature and pH (Glick et al., 2014). Equally important seems to be an exceptionally good water solubility, no mutagenic properties and its invulnerability to efflux mechanisms, what was seen in *S. cerevisiae* (Lis et al., 2012) and other fungi (Dyląg et al., unpublished data). Moreover, 3BP can be conveniently administered orally or intravenously (El Sayed et al., 2014; Azevedo-Silva et al., 2016; Lis et al., 2016b), and its effect can be reversible and easily monitored and controlled by GSH supplementation (Lis et al., 2016a; El Sayed et al., 2017).

The results from this study underline the high *in vitro* activity of 3BP against the *Prototheca* algae, its synergistic effect when used in combination with AMB, and the safety of the drug toward the mammalian cells tested. Given its advantageous physico-chemical and pharmacokinetic properties, 3BP constitutes a new promising option for the treatment of protothecal disease. However, to better explore the potential of 3BP against *Prototheca* spp., more studies involving both *in vitro* and *in vivo* approaches, are needed.

## Author contributions

TJ and MD: designed all experiments, supervised the entire work, performed data analysis, and wrote the manuscript; KR and KN: performed cytotoxicity and 3BP transport assays, respectively. All authors read and approved the submitted version of this work.

### Conflict of interest statement

The authors declare that the research was conducted in the absence of any commercial or financial relationships that could be construed as a potential conflict of interest.
